# Genomic Study of Cardiovascular Continuum Comorbidity

**Published:** 2015

**Authors:** O. A. Makeeva, A. A. Sleptsov, E. V. Kulish, O. L. Barbarash, A. M. Mazur, E. B. Prokhorchuk, N. N. Chekanov, V. A. Stepanov, V. P. Puzyrev

**Affiliations:** Research Institute of Medical Genetics, Nab. Ushayki, 10, Tomsk, 634050, Russia; Research Institute for Complex Issues of Cardiovascular Diseases, Sosnovy Blvd., 6, Kemerovo, 650000, Russia; Genoanalitika, Leninskie Gory, 1/77, Off. 102, Moscow, 119234, Russia; Siberian State Medical University, Moskovskiy Trakt, 2, Tomsk, 634050, Russia

**Keywords:** genetic polymorphism, multifactorial diseases, syntropy, comorbidity, association studies, cardiovascular continuum

## Abstract

Comorbidity or a combination of several diseases in the same individual is a common and widely investigated phenomenon. However, the genetic background for non–random disease combinations is not fully understood. Modern technologies and approaches to genomic data analysis enable the investigation of the genetic profile of patients burdened with several diseases (polypathia, disease conglomerates) and its comparison with the profiles of patients with single diseases. An association study featuring three groups of patients with various combinations of cardiovascular disorders and a control group of relatively healthy individuals was conducted. Patients were selected as follows: presence of only one disease, ischemic heart disease (IHD); a combination of two diseases, IHD and arterial hypertension (AH); and a combination of several diseases, including IHD, AH, type 2 diabetes mellitus (T2DM), and hypercholesterolemia (HC). Genotyping was performed using the “My Gene” genomic service (www.i–gene.ru). An analysis of 1,400 polymorphic genetic variants and their associations with the studied phenotypes are presented. A total of 14 polymorphic variants were associated with the phenotype “IHD only,” including those in the *APOB*, *CD226*, *NKX2–5, TLR2*, *DPP6*, *KLRB1*, *VDR*, *SCARB1*,* NEDD4L*, and *SREBF2 *genes, and intragenic variants rs12487066, rs7807268, rs10896449, and rs944289. A total of 13 genetic markers were associated with the “IHD and AH” phenotype, including variants in the *BTNL2, EGFR*, *CNTNAP2*, *SCARB1*, and *HNF1A *genes, and intragenic polymorphisms rs801114, rs10499194, rs13207033, rs2398162, rs6501455, and rs1160312. A total of 14 genetic variants were associated with a combination of several diseases of cardiovascular continuum (CVC), including those in the *TAS2R38*, *SEZ6L*, *APOA2, KLF7*, *CETP*,* ITGA4*, *RAD54B*, *LDLR*, and *MTAP *genes, along with intragenic variants rs1333048, rs1333049, and rs6501455. One common genetic marker was identified for the “IHD only” and “IHD and AH” phenotypes: rs4765623 in the* SCARB1 *gene; two common genetic markers, rs663048 in *SEZ6L *and intragenic rs6501455, were identified for the “IHD and AH” phenotype and a combination of several diseases (syntropy); there were no common genetic markers for the “syntropy” and “IHD only” phenotypes. Classificatory analysis of the relationships between the associated genes and metabolic pathways revealed that lipid–metabolizing genes are involved in the development of all three CVC variants, whereas immunity-response genes are specific to the “IHD only” phenotype. The study demonstrated that comorbidity presents additional challenges in association studies of disease predisposition, since the genetic profile of combined forms of pathology can be markedly different from those for isolated “single” forms of a disease.

## INTRODUCTION

Multiple, concurrent diseases have long been an issue in clinical practice [1, 2]. In developed countries, up to 80% of the healthcare budget is spent on patients with four or more diseases [3]. The most common term for this phenomenon is “comorbidity” [1]. However, only a portion of concurrent diseases with a common genetic basis and similar pathogenesis is referred to as “syntropies” or “attraction diseases” [4]. There are many clinically proven syntropic diseases, e.g., immunodependent diseases (allergic and autoimmune) [5, 6]; endocrine system diseases, including the combination of diabetes mellitus (T2DM), autoimmune thyroiditis, and the celiac disease [7]; some forms of mental illness [8]. Among them are cardiovascular diseases (CVDs), which are related to the concept of cardiovascular continuum (CVC).

The term “cardiovascular continuum” was proposed by Dzau and Braunwald in the early 1990s. The concept of CVC describes the development and progression of a disease over time and also reflects the essence of the relationships among various risk factors (genetic and environmental), highlighting their integrity [9-11]. The CVC hypothesis postulates that cardiovascular diseases (CVDs) are a specific chain of events that is triggered by numerous, interrelated or independent risk factors, progresses as a result of the activation of multiple signaling pathways and physiological processes, and, ultimately, leads to the end–stage heart disease. Cardiovascular risk factors include high cholesterol, arterial hypertension, diabetes mellitus, smoking, obesity, and physical inactivity. Cardiovascular disease continuum (continuum of clinical phenotypes) is based on the pathophysiologic continuum that includes progressive molecular and cellular changes manifesting themselves as a disease at the clinical level. Fundamentally, these processes are based on oxidative stress and endothelial dysfunction that, in turn, initiate a cascade of events, including disturbances in the system of vasoactive mediators, non–specific inflammatory response, and vascular remodeling. All the phenomenons mentioned above result in damage to target organs.

In clinical practice, comorbidity (combination of diseases) makes the use of genomic markers for predicting the risk of a disease more difficult. Do genetic variants that increase the risk of a certain single disease play the same pathogenic role in the case of complex phenotypes (combinations of diseases) or does their contribution change? How to account for the genetic pleiotropy and diverse effects of some genetic variants during the development of approaches to genetic testing? A genetic variant may be a risk factor for one disease and a protective factor for another.

This paper presents the results of a comparative analysis of a genetic component of three clinical phenotypes (a single disease, a combination of two diseases, and a combination of several CVDs) using a set of genomic markers provided by the “My gene” service (www.i–gene.ru). The main objective of the study was to identify common and specific genetic markers and perform a comparative analysis of the genetic component of various combinations of cardiovascular diseases.

## MATERIALS AND METHODS

The study included three groups of patients with various combinations of CVDs and a control sample of relatively healthy individuals. Patients with various combinations of diseases were selected from a population of more than 800 patients admitted to a specialized cardiology hospital for acute coronary syndrome (ACS). All patients underwent a detailed clinical and laboratory examination for both primary diagnosis and concurrent pathologies. The inclusion criteria for the first sample group were as follows: patients with IHD (myocardial infarction) without any comorbidity (*n *= 61). This group of patients was diagnosed with IHD only, while other diseases, such as AH and T2DM, were excluded. The second sample group included patients with a combination of two diseases: IHD and AH (*n *= 180); patients with any other CVDs were excluded. The third sample group included patients with IHD, AH, T2DM, and HC (*n *= 68). The sample group with a combination of several diseases is hereafter referred to as “CVC syntropy.” The remaining patients with a combination of IHD and some other pathology were excluded from the study.

The control group of relatively healthy individuals (*n *= 131) included subjects without CVD in their medical history, with normal blood pressure, and normal echocardiographic and lipid profile parameters. On the basis of these criteria, individuals of this group were selected from an epidemiologicaly collected sample formed for studying IHD risk factors.

Genomic DNA was isolated by standard method of phenol–chloroform extraction [12]. Genotyping was performed using Illumina Custom Genotyping Microarrays iSelectHD microarrays produced by an order of Genoanalitika for the “My gene” genomic service. A microarray comprised 4,416 genetic variants; of them, 2,121 were single nucleotide substitutions in 98 genes of monogenic diseases, 1,913 were polymorphic variants of the nuclear genome, and 382 were polymorphic variants of the mitochondrial genome.

In order to minimize technological errors during genotyping, polymorphic variants and DNA samples were collected using the following criteria: 1) proportion of genotyped single nucleotide variants in one sample should be no less than 98%; 2) proportion of genotyped samples for each polymorphic variant should be more than 98%; 3) genotype identity of any two samples should be less than 98%; 4) compliance of genotypic data of gender identity with an individual’s gender; 5) compliance with the Hardy–Weinberg equilibrium in a pooled sample at a statistically significance level of P > 10–8and in the control group at P > 0.05; 6) frequency of a rare allele is more than 5%; and 7) polymorphic variants are localized in autosomes.

A total of 407 genomic DNA samples and 1,400 polymorphism variants were selected for further analysis after genotyping quality control with subsequent exclusion of single nucleotide substitutions of monogenic disease genes and single nucleotide variants localized in sex chromosomes and mitochondria.

GenABEL software packages for the statistical environment R (version 2.14.2) were used to analyze associations. The statistical significance level calculated by random permutations with 10,000 replications (permutation test) was considered to be equal to P < 0.05. The network analysis of intergenic interactions was performed using the Search Tool for the Retrieval of the Interacting Genes/Proteins platform [13]. The WEB– based Gene Set Analysis Toolkit was used for the annotation of metabolic and signaling pathways [14]. The predictive efficiency of the polymorphic variants that demonstrated a statistically significant association with the studied phenotypes was analyzed by standard AUC (Area Under Curve) calculations.

## RESULTS

The main objective of the study was to identify common and specific genes for the studied phenotypes: a single disease (IHD), a combination of two diseases (IHD and AH), and a complex “syntropy” phenotype, which refers to a combination of several cardiovascu lar diseases. To identify the genes associated with a particular phenotype (disease or combination of diseases), we compared the frequencies of the alleles and genotypes in patients and in the control group (case– control study), calculated odds ratios (OR), and evaluated the prognostic significance of genetic markers with statistically significant associations. All genetic markers/genes were classified according to association with a particular pathway or class of genes. The WebGestalt (WEB–based GEne SeT AnaLysis Toolkit) service was used to annotate signaling and metabolic pathways.

*Tables 1–3 *show the nomenclature and key statistics of the genetic variants whose allele frequencies were different in the study and control group: chromosomal localization, rs–number, rare allele frequency, location relative to the nearest genes, and OR value. *Table 4* shows predictive values of the polymorphic variants associated with a certain phenotype: disease or disease combination.

Below, we present the results obtained for each of the studied phenotypic groups.

**Genetic markers associated with the IHD phenotype**

**Table 1:** Polymorphic variants associated with the “IHD only” phenotype

Chromosome	Gene abbreviation	SNP Localization	SNP	Allele: MAF	Allele: OR (95% CI)	Fisher’s exact test	Genotypes: OR (95% CI)	Pχ^2^ perm.test
2p24	APOB	missense	rs1367117	A:0.29	A:1.76 (1.08–2.88)	0.022	AA:3.59 (1.25–10.85)	0.01
3q13.1			rs12487066	G:0.23	A:0.47 (0.28–0.79)	0.0026	AA:0.37 (0.18–0.74)	0.0031
4q32	TLR2	intron	rs1898830	G:0.34	A:0.51 (0.31–0.82)	0.0038	AA:0.43 (0.19–0.9)	0.021
5q34	NKX2–5	5’–ends	rs3095870	A:0.39	G:1.83 (1.1–3.12)	0.017	GG:2.09 (1.06–4.17)	0.024
7q36.1			rs7807268	C:0.45	C:1.96 (1.22–3.17)	0.0045	CC:2.73 (1.29–5.82)	0.0065
7q36.2	DPP6	intron	rs10239794	A:0.37	A:1.99 (1.24–3.2)	0.0029	AA:3.35 (1.4–8.26)	0.0041
11q13			rs10896449	G:0.46	A:0.57 (0.35–0.92)	0.016	AA:2.36 (1.16–4.8)	0.011
12p13	KLRB1	intron	rs4763655	A:0.34	G:0.56 (0.35–0.91)	0.014	GG:0.45 (0.21–0.94)	0.031
12q13.11	VDR	intron	rs7975232	A:0.54	A:0.57 (0.35–0.91)	0.013	AA:0.32 (0.11–0.8)	0.009
12q24.31	SCARB1	intron	rs4765623	A:0.25	G:0.57 (0.35–0.95)	0.025	GG:0.4 (0.2–0.8)	0.0068
14q13.3			rs944289	G:0.38	A:0.55 (0.34–0.88)	0.012	AA:0.42 (0.18–0.9)	0.017
18q22.3	CD226	missense	rs763361	A:0.39	A:1.79 (1.12–2.87)	0.012	AA:2.51 (1.11–5.7)	0.018
18q21	NEDD4L	intron	rs3865418	A:0.53	A:0.56 (0.35–0.91)	0.013	AA:0.36 (0.13–0.86)	0.017
22q13	SREBF2	intron	rs2267439	G:0.39	A:2.25 (1.33–3.92)	0.0018	AA:2.32 (1.17–4.67)	0.01

Note. Here and in Tables 2 and 3, MAF is the minor (rare) allele frequency; CI is the confidence interval; OR (95% CI) is the odds ratio (95% confidence interval); Pχ^2^ is the level of significance of the chi–square test for an allele with one degree of freedom; Pχ^2^ permutation test is the level of significance of the chi–square test for genotypes with two degrees of freedom.

A total of 14 polymorphic variants are associated with the “IHD only” phenotype (*Table 1*). Of them, two are missense substitutions: rs1367117 in the *APOB *gene and rs763361 in the *CD226 *gene. One, rs3095870, is located near the 5’–end of the *NKX2–5 *gene. Seven variants are located in intronic regions: rs1898830 in the* TLR2 *gene, rs10239794 in *DPP6*, rs4763655 in *KLRB1*, rs7975232 in *VDR*, rs4765623 in *SCARB1*, rs3865418 in *NEDD4L*, and rs2267439 in *SREBF2*. Four genetic markers are located in intergenic spaces: rs12487066, rs7807268, rs10896449, and rs944289 (*Table 1*). The prognostic value of markers associated with this phenotype varied from 0.62 for rs12487066 to 0.57 for rs1367117 (*Table 4*).

**Table 2:** Polymorphic variants associated with the “IHD and AH” phenotype

Chromosome	Gene abbreviation	SNP Localization	SNP	Allele: MAF	Allele: OR (95% CI)	Fisher’s exact test	Genotypes: OR (95% CI)	Pχ^2^ perm.test
1q42			rs801114	C:0.39	C:0.66 (0.46–0.95)	0.025	CC:0.3 (0.12–0.7)	0.0018
2q35	XRCC5	3’–end	rs2440	A:0.33	A:1.57 (1.1–2.26)	0.012	AA:2.48 (1.12–5.93)	0.017
6p21.3	BTNL2	missense	rs2076530	G:0.39	G:1.57 (1.1–2.23)	0.01	GG:1.96 (1.02–3.92)	0.034
6q23			rs10499194	A:0.35	G:1.52 (1.04–2.22)	0.026	GG:2.07 (1.25–3.44)	0.0037
6q23			rs13207033	A:0.35	G:1.49 (1.02–2.18)	0.033	GG:2.02 (1.22–3.36)	0.004
7p12	EGFR	intron	rs763317	A:0.42	G:0.67 (0.47–0.95)	0.022	GG:0.5 (0.29–0.88)	0.011
7q35	CNTNAP2	intron	rs7794745	T:0.36	A:0.69 (0.48–0.98)	0.032	AA:0.52 (0.31–0.87)	0.011
12q24.31	SCARB1	intron	rs4765623	A:0.25	A:0.63 (0.43–0.93)	0.017	AA:0.49 (0.3–0.82)	0.0038
15q26			rs2398162	G:0.4	A:0.68 (0.47–0.97)	0.033	AA:0.43 (0.19–0.93)	0.027
17cen–q21.3	HNF1A	intron	rs4430796	G:0.36	G:1.59 (1.12–2.28)	0.0077	GG:2.12 (1.09–4.3)	0.024
17q24.3			rs6501455	G:0.32	A:1.52 (1.05–2.19)	0.023	AA:2.4 (1.08–5.75)	0.024
20p11			rs1160312	G:0.42	G:1.56 (1.1–2.22)	0.011	GG:2.21 (1.16–4.38)	0.013
22q12.1	SEZ6L	missense	rs663048	A:0.19	C:0.57 (0.38–0.87)	0.0082	CC:0.53 (0.31–0.88)	0.011

Note. See Table 1 note.

On the basis of their primary biological function in the body, several groups of associated genes were selected to analyze the structure of a hereditary component of the studied phenotypes. For example, gene variants associated with the “IHD only” phenotype can be arbitrarily subdivided **into three groups**: 1) genes responsible for the lipid metabolism, 2) genes associated with immunity, and 3) genes specific to the cardiac function.

**Table 3:** Polymorphic variants associated with the “CVC syntropy” group

Chromosome	Gene abbreviation	Localization	SNP	Allele: MAF	Allele: OR (95% CI)	Fisher’s exact test	Genotypes: OR (95% CI)	Pχ2 perm. test
1q21	APOA2	5’–end	rs5082	G:0.47	A:1.83 (1.15–2.93)	0.0082	AA:2.42 (1.22–4.82)	0.007
2q31.3	ITGA4	synonymous substitution	rs1143674	A:0.39	A:1.76 (1.12–2.78)	0.011	AA:2.84 (1.27–6.47)	0.0069
2q32	KLF7	5’–end	rs7568369	A:0.28	C:0.45 (0.28–0.73)	0.00084	CC:0.34 (0.16–0.68)	0.0007
7q34	TAS2R38	missense	rs1726866	G:0.48	A:1.65 (1.04–2.63)	0.028	AA:2.42 (1.22–4.82)	0.0073
		missense	rs10246939	G:0.48	A:1.65 (1.04–2.63)	0.028	AA:2.42 (1.22–4.82)	0.0073
8q22.1	RAD54B	synonymous substitution	rs2291439	G:0.42	G:0.6 (0.37–0.97)	0.032	GG:0.25 (0.06–0.77)	0.011
9p21	MTAP	intron	rs7023329	G:0.52	A:1.95 (1.23–3.11)	0.0031	AA:2.38 (1.2–4.76)	0.0079
9p21.3			rs1333048	C:0.45	A:0.6 (0.38–0.94)	0.022	AA:0.37 (0.15–0.87)	0.018
9p21.3			rs1333049	G:0.44	C:0.61 (0.38–0.95)	0.028	CC:0.39 (0.16–0.88)	0.022
16q21	CETP	5’–end	rs183130	A:0.35	G:2.04 (1.21–3.51)	0.006	GG:2.19 (1.13–4.32)	0.013
17q24.3			rs6501455	G:0.32	G:2.06 (1.3–3.29)	0.0015	GG:3.91 (1.56–10.33)	0.0016
19p13.2	LDLR	intron	rs2738446	G:0.34	C:0.61 (0.38–0.97)	0.032	CC:0.38 (0.18–0.77)	0.0045
		synonymous substitution	rs688	A:0.34	G:0.61 (0.38–0.97)	0.032	GG:0.38 (0.18–0.77)	0.0048
22q12.1	SEZ6L	missense	rs663048	A:0.19	C:0.52 (0.31–0.88)	0.01	CC:0.49 (0.25–0.95)	0.027

Note. See Table 1 note.

The group of genes involved in the **lipid metabolism** includes *APOB*, *SREBF2*, and *SCARB1*. For example, the *APOB *gene product is the main apolipoprotein of chylomicron and LDLs. Polymorphic variants and some mutations of the *APOB *gene are known to be associated with HC and a high risk of IHD [15, 16].

**Table 4:** Predictive efficiency of polymorphic variants associated with the “IHD only,” “IHD and AH,” and “CVC syntropy” phenotypes

“IHD only”		“CVC syntropy”		“IHD and AH”	
SNP	AUC	SNP	AUC	SNP	AUC
rs1367117	0.57	rs801114	0.56	rs5082	0.60
rs12487066	0.62	rs2440	0.55	rs1143674	0.59
rs1898830	0.59	rs2076530	0.55	rs7568369	0.63
rs3095870	0.59	rs10499194	0.59	rs1726866	0.60
rs7807268	0.60	rs13207033	0.59	rs10246939	0.60
rs10239794	0.59	rs763317	0.57	rs2291439	0.57
rs10896449	0.60	rs7794745	0.58	rs7023329	0.60
rs4763655	0.61	rs4765623	0.59	rs1333048	0.58
rs7975232	0.59	rs2398162	0.55	rs1333049	0.58
rs4765623	0.61	rs4430796	0.56	rs183130	0.60
rs944289	0.59	rs6501455	0.58	rs6501455	0.59
rs763361	0.58	rs1160312	0.56	rs2738446	0.60
rs3865418	0.59	rs3843763	0.58	rs688	0.60
rs2267439	0,60	rs663048	0,48	rs663048	0,59

Note. AUC is the area under the curve.

The *SREBF2 *gene product is a transcriptional activator required to maintain lipid homeostasis. In particular, this gene regulates the *LDLR *gene, which encodes the LDL receptor and also affects the cholesterol level and synthesis of fatty acids [17].

The *SCARB1 *gene encodes a receptor of the various ligands involved in the lipid metabolism, including phospholipids, cholesterol esters, lipoproteins, and phosphatidylserine. Presumably, the product of this gene is also involved in the phagocytosis of apoptotic cells via its phosphatidylserine–binding activity, as well as in the uptake of cholesterol esters by HDLs [18].

The group of genes associated with **immunity **regulation includes *TLR2*, *KLRB1*, *CD226*, and *VDR*. The* TLR2 *gene interacts with the *LY96 *and *TLR1 *genes and plays an important role in the formation of the innate immune response to bacterial lipoproteins and other components of the bacterial wall. It is activated by the MYD88 and TRAF6 factors, which leads to the activation of cytokines by NF–kB and an inflammatory response. The product of this gene may also play a role in apoptosis [19]. Structural polymorphisms in this gene are associated with susceptibility to leprosy and some infectious diseases [20].

The *KLRB1 *gene inhibits natural killer cells (cytotoxicity cells). It is expressed in T–lymphocytes of peripheral blood, primarily in T–cells with antigenic memory [21].

The *CD226 *gene encodes a receptor involved in intercellular adhesion, lymphocyte signaling, and cytotoxicity and secretion of lymphokines by cytotoxic T– lymphocytes and NK–cells [22].

The *VDR *gene encodes the nuclear vitamin D3 receptor; however, this protein is well known to function also as a receptor of secondary bile acid, lithocholic acid. The vitamin D3 receptor is involved in the mineral metabolism (calcium exchange), although it may also regulate a number of other metabolic pathways; in particular, the immune response [23].

The third group includes genes associated with various metabolic and signaling pathways; however, these genes are involved in specific cardiac functions. Two of them are associated with the transmembrane transport of electrolytes and the **cardiac conduction system** (*NEDD4L *and *DPP6*), and the third gene, *NKX2–5*, encodes the heart–specific **transcription factor**.

The *NEDD4L *gene plays an important role in the epithelial sodium transport via control of the expression of sodium channels on epithelial cells surface. The product of this gene was shown to be involved in the formation and transmission of the membrane potential along the cardiac conduction system [24]. Dunn *et al *[25] have demonstrated the association of some *NEDD4L* polymorphic variants with essential hypertension. The A–allele of the rs3865418 polymorphism is associated with high diastolic pressure in the Chinese population (*OR *= 1.31 (1.04–1.67), *P *= 0.025) [26]. In the present study, the A–allele and the AA genotype demonstrated a protective effect against IHD (*Table 2*).

The *DPP6 *gene encodes a membrane protein (dipeptidyl aminopeptidase–likeprotein 6) which is a member of the S9B serine protease family. It can bind to specific voltage–dependent potassium channels, thereby affecting their expression, biophysical properties, and channel activity [27]. Defects in the *DPP6 *gene lead to familial paroxysmal ventricular fibrillation type 2 [28].

The *NKX2–5 *gene, which is expressed exclusively in the heart, encodes the homeobox–containing transcription factor. This factor is directly involved in the formation and development of the heart *in utero *[29]. Mutations in the *NKX2–5 *gene cause various heart defects: from small anomalies to Fallot’s tetralogy (MIM:108900,187500).

**Genetic markers associated with the “IHD and AH” phenotype**

Among the studied genetic markers, 13 were associated with the “IHD and AH” phenotype (*Table 2*). Two of them are missense substitutions (rs2076530 in the* BTNL2 *gene and rs663048 in the *SEZ6L *gene), four are variants in introns (rs763317 in *EGFR*, rs7794745 in *CNTNAP2*, rs4765623 in *SCARB1*, rs4430796 in *HNF1A*), and six are located in intergenic regions (rs801114, rs10499194, rs13207033, rs2398162, rs6501455, and rs1160312).

The predictive value of the genetic markers evaluated by the AUC parameter (area under the ROC–curve) varied from 0.59 to 0.55 (*Table 4*).

Notably, it is difficult to associate this set of genes with any particular pathway and to classify it in a similar way as for the genes associated with the “IHD only” phenotype. However, it is noteworthy that several genes are associated with **immunity and susceptibility to cancer and radiosensitivity**.

For example, the *BTNL2 *gene is a regulator of immunity: its product, the butyrophilin–like protein 2, belongs to the family of B7 receptors, which function as T–cell–stimulating molecules by affecting the production of cytokines and regulating T–cell proliferation. Polymorphic variants of this gene are associated with an increased risk of prostate cancer, Kawasaki disease, as well as damage to coronary arteries in this disease [30]. The association of variants of this gene with susceptibility to tuberculosis was demonstrated [31]. According to the data of a genome–wide study [32], variations in *BTNL2 *are also associated with the development of coronary atherosclerosis.

Genes in some way associated with the oncogenesis and immune system include *XRCC5*, *EGFR*, *HNF1A*, and *SEZ6L*. The *XRCC5 *gene encodes an 80 kDa subunit of the Ku protein. The heterodimeric protein Ku is an ATP–dependent DNA helicase II, which is involved in DNA repair by non–homologous end joining. The Ku protein is involved in the recombination required to generate a diversity of antigen–binding sites of antibodies in mammals. In addition, Ku–proteins are involved in telomeric length maintenance and telomeric silencing [33]. A rare microsatellite polymorphism of this gene is known to be associated with oncopathology and radiosensitivity.

A protein encoded by the *EGFR *gene is a transmembrane glycoprotein, the epidermal growth factor receptor [34]. Defects in this gene lead to disruption of apoptosis; the association of this gene with carcinogenesis has been extensively studied [35]. The A–allele of rs763317, which is considered to be a lung cancer risk allele, is adverse to the “IHD and AH” phenotype.

The *HNF1A *gene product is a transcriptional activator that regulates the tissue–specific expression of several genes, particularly in pancreas and liver cells [36]. Defects in this gene lead to the familial form of liver adenomas (MIM:142330), MODY3–type diabetes mellitus (MIM:600496), and insulin–dependent type 2 diabetes mellitus (MIM:612520). We demonstrated that the G– allele, which is a T2DM risk factor, is unfavorable to the development of the combined IHD and AH pathology.

The *SEZ6L *gene function is poorly understood. It is assumed to be associated with specific functions of the endoplasmic reticulum. The gene has been extensively studied, because it is expressed in the brain tissue and lungs but not in lung cancer cells [37]. Data on an association between polymorphic variants of *SEZ6L* and IHD were reported [38], but the mechanism of this relationship is not known. Our data demonstrate that the lung cancer risk allele is also an unfavorable factor for IHD in combination with AH.

We found that the *CNTNAP2 *gene is associated with the cardiovascular phenotype (which is unusual, given the known functions of the gene). The *CNTNAP2* gene encodes a transmembrane protein belonging to the family of neurexins, which act as cell–adhesion molecules and receptors in the nervous system. The CNTNAP2 protein performs its major functions in myelinated axons, enabling the interaction between neurons and glia. It is also responsible for the localization of potassium channels and differentiation of axons into separate functional subdomains. *CNTNAP2 *gene variants are associated with a wide range of mental disorders, including autism, schizophrenia, mental retardation, dyslexia, and disruption of language functions [39]. The A–allele is a risk factor for schizophrenia but is a protective factor in the case of the “IHD and AH” phenotype.

As we have indicated, the *SCARB1 *gene is associated with lipid metabolism. This gene is “common” for the “IHD” and “IHD and AH” phenotypes.

The functions of several genetic variants located in intergenic regions require further analysis. An association with a disease may be explained by a linkage disequilibrium with some other genes/variants directly involved in the formation of the disease or by an independent regulatory action. According to some studies, the intergenic variant rs1160312A is associated with hair loss, but at the same time, it is protective in the case of IHD combined with AH. According to a Wellcome Trust large–scale study conducted using the “case–control” approach, the A–allele of rs2398162 of this gene is a risk factor for essential hypertension; in our work, however, this allele is protective.

**Genetic markers associated with the “CVC syntropy” phenotype**

A total of 14 markers are associated with CVC syntropy (*Table 3*). Of these, three are missense substitutions: rs1726866 and rs10246939 in the *TAS2R38* gene and rs66048 in the *SEZ6L *gene; three are located near the 5’–region of genes: rs5082 of the *APOA2* gene, rs7568369 in the 5’–region of the *KLF7 *gene, and rs183130 in the 5’–region of the *CETP *gene; three variants are synonymous substitutions: rs1143674 in the* ITGA4 *gene, rs2291439 in the *RAD54B *gene, and rs688 in the *LDLR *gene; two variants are located in intronic regions: rs2738446 in the *LDLR *gene and rs7023329 in the *MTAP *gene; three markers are located in intergenic spaces: rs1333048 and rs1333049 in the 9p21.3 region and rs6501455 localized in 17q24.3 between the *KCNJ2* and *SOX9 *genes, at approximately the same distance (*ca. *1 million bp).

The genes associated with the “CVC syntropy” phenotype can be assigned to one of the following groups: a) responsible for **lipid metabolism impairment**, b) genes of **immunity and inflammation**, c) **genes with miscellaneous functions**.

The genes *CETP*, *LDLR*, and *APOA2 *are associated with the **lipid metabolism**. The *CETP *gene encodes a transporter of insoluble cholesterol esters. *CETP *polymorphic variants affect the level of high density lipoproteins (HDLs). In particular, mutations in this gene are associated with the development of hyper–α–lipoproteinemia, accompanied by high HDL levels (MIM:143470). The G–allele of rs183130 is associated with a low level of cholesterol in HDLs [40]. We demonstrated its involvement in the formation of the “CVC syntropy” phenotype.

The *LDLR *gene encodes a protein that functions as an intermediary of endocytosis of cholesterol–rich LDLs [41]. Variants of the *LDLR *gene, rs2738446 and rs688, are in linkage disequilibrium. In the Framingham study, rs688 is associated with IHD but is not directly related to such an important endophenotype as the level of lipoproteins [42]. According to some studies, rs688 in the *LDLR *gene may be associated with the onset of IHD via modulation of the coagulation factor VIII activity [43].

The *APOA2 *gene encodes a HDL protein particle. Mutations in *APOA2 *cause familial HC [44]. According to some reports, the AA genotype of rs5082 in the* APOA2 *gene is associated with a high risk of IHD in males. In our work, the AA genotype was associated with a high risk of the “CVC syntropy” phenotype.

On the basis of their main functions, the genes* ITGA4*, *MTAP*, and *CDKN2V *may be assigned to a group of **immunity and inflammation **genes. The* ITGA4 *gene encodes a protein of the integrinα–chain. Integrins are the most important intercellular adhesion molecules. They are heterodimeric membrane receptors consisting of α– and β–chains and functioning as cell–substrate and cell–cell adhesion receptors. Increased adhesion is known to be important in endothelial dysfunction in the case of inflammation, arteriosclerosis, and other pathological processes [45, 46]. There are no data on the association of rs1143674 in the* ITGA4 *gene with cardiovascular phenotypes; however, it is known that this variant is associated with alternative splicing. The rs1143674 A–allele is associated with an increased risk of autism [47].

*The MTAP *gene is located in close proximity to *CDKN2A/ 2B *[48]. The *MTAP *and *CDKN2B *genes were shown to be expressed in cells and tissues involved in the development of atherosclerosis, including endothelial cells, macrophages, and smooth muscle cells of coronary arteries [49]. The *MTAP *gene was reported to be capable of acting as a tumor growth suppressor [50].

Four other genes, *RAD54B*, *SEZ6L*, *TAS2R38, *and* KLF7 *are genes with miscellaneous functions. The product of *RAD54B *is involved in DNA repair and mitotic recombination. Mutations in this gene may be a cause of colon cancer and lymphomas [51]. Data on the functional role of the rs2291439 variant are limited, and there is no information on an association between this marker and diseases of the cardiovascular system.

The *TAS2R38 *gene encodes a receptor responsible for bitter taste reception [52]. *TAS2R38 *gene haplotypes define up to 85% of the variance in the sensitivity to flavors, from bitter to sweet [53]. Carriership of certain *TAS2R38 *variants affects food preferences, for example, for carbohydrate–rich or lipid–rich food, which is considered a risk factor for the development of metabolic disorders and CVDs. Alleles of the gene that are associated with the syntropy phenotype are included in the haplotype responsible for the inability to feel the bitter taste; the same alleles are associated with the development of T2DM.

The *KLF7 *gene product belongs to a group of transcriptional activators and is expressed in many body tissues. A study of animal models demonstrated that* KLF7 *specifically regulates the expression of *TrkA*, which encodes the neurotrophic tyrosine kinase receptor type 1. A nonsense mutation of the *KLF7*gene leads to disruption of many nociceptive sensory receptors [54]. Investigation of *KLF7 *gene polymorphisms revealed an association with T2DM risk in the Japanese population and a protective effect against obesity in the Danish population [55, 56].

Characterization of the *SEZ6L *gene is provided above, since this gene is associated with the “IHD and AH” phenotype.

The functional significance of polymorphisms associated with the studied phenotype and located in intergenic regions remains unknown. According to [57], rs6501455 is associated with prostate cancer, but it is a protective allele for CVC syntropy. This gene is located on chromosome 17, *ca. *1 million bp away from the* SOX9 *and *CALM2P1 *genes, with approximately more than 40,000 SNPs being identified in this same region. Importantly, almost all polymorphisms in this genomic region, which are present in the PubMed database, are associated with a particular CVD.

## DISCUSSION

**Fig. 1:**
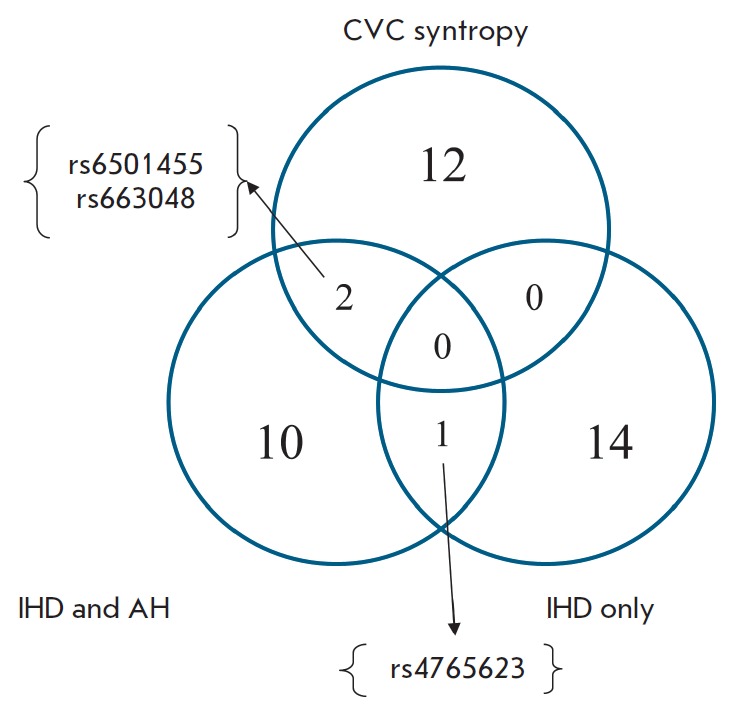
Venn diagram of genetic markers associated with the studied phenotypes: common and specific variants

The objective of this study was to identify common and specific genes for three phenotypes: a single disease, a combination of two diseases, and a combination of CVDs. *Fig. 1*shows the number of genes that are common and specific to the three phenotypes. For example,** two common genes **were identified for syntropy (a combination of several diseases: IHD, AH, T2DM, and HC in this study) and a combination of two diseases (IHD and AH); **one common gene **was identified between the “IHD and AH” and “IHD only” phenotypes; whereas there were **no common genes **between “IHD only” and “CVC syntropy” among the studied genes. A total of 14 specific genetic variants unique to the “IHD only” phenotype were identified: 12 were unique to “CVC syntropy,” and 10 were unique to “IHD and AH.”

In the case of comorbidities, it is important to know not only the proportion of common and specific genes, but also the profile of associated variants (their physiological role in the body), since the profile may indicate the most important pathways involved in the development of a pathology and define approaches to treatment. As seen from the associative profile provided for each studied phenotype, the “IHD only” phenotype is characterized by genes that regulate the lipid metabolism, genes associated with immunity, and genes specific to the heart function, such as the genes that control the cardiac conduction system.

Examination of the profile for a combination of two diseases, one of which is IHD, reveals a markedly different picture, and the gene variants associated with this phenotype seem rather unexpected; it is hard to relate a set of genes to a particular pathway and classify them similarly to the genes associated with the “IHD only” phenotype. Notably, several genes of immunity and predisposition to cancer are associated with the phenotype of a two–disease combination.

The genetic profile for the CVC syntropy phenotype appears to be logical in general; it includes genes responsible for lipid metabolism impairment and genes that control the immunity and inflammatory response (and genes with miscellaneous functions).

It should be noted that our analysis of a wide panel of markers (1,400) associated, according to the published data, with widespread diseases revealed previously unknown relationships (as it usually occurs in genome– wide studies); e.g., the association of the genes of cancer and neurological and psychiatric diseases with cardiovascular phenotypes.

**Fig. 2:**
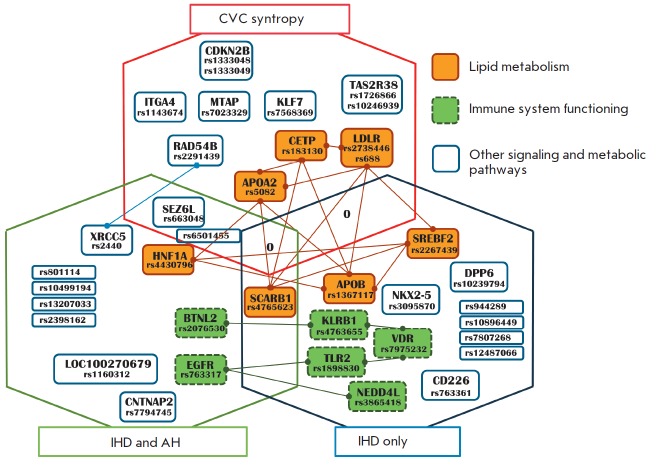
Venn diagram of the relationships between all phenotype–associated genes and polymorphic variants with allowance for their association with a functional class. Intergenic relationships were developed based on the data of the SearchTool for the Retrieval of Interacting Genes/Proteins (STRING) online service

A descriptive analysis of the functions of associated genes is supplemented by a classificatory network analysis of intergenic interactions that enables to retrace interaction chains for a set of genes, a STRING–analysis (*Fig. 2*). This analysis allows a formal assignment of particular genes to the most important metabolic pathways. This formal analysis revealed that the genes associated with immune function and lipid metabolism genes predominate among the IHD genes. In the case of the IHD and AH phenotype, two genes were related to the immune system, and two genes were associated with lipid metabolism. That is the *SCARB1 *lipid metabolism gene that is common for these two pathologies. Among the CVC sintropy–associated genes, three genes were identified as genes related to lipid metabolism. According to the STRING analysis, the remaining genes were not related to a specific metabolic pathway.

## CONCLUSIONS

Our findings indicate that lipid metabolism genes are involved in the formation of all variants of diseases (including various combinations) of the cardiovascular continuum, while the regulatory genes of the immune system are specific to IHD and do not participate in the development of CVC syntropy.

Coming back to the discussion of the role of syntropy (non–random combination of diseases) and the widespread phenomenon of comorbidity, we identified an additional complexity in the use of the data of genetic association studies in practical (diagnostic) tests for predisposition to common diseases. The genetic profile for a combination of several diseases can be markedly different from the profile for isolated forms. Identification of syntropic genes (influencing the development of a complex syntropyphenotype) is of interest not only for diagnostic purposes, but also, first and foremost, for predicting (or explaining present facts) the effect of some medications in the case of several, concurrent diseases.

## Abbreviations

AH: arterial hypertension
HC: hypercholesterolemia
IHD: ischemic heart disease
HDLs: high density lipoproteins
LDLs: low density lipoproteins
ACS: acute coronary syndrome
T2DM: type 2 diabetes mellitus
CVD: cardiovascular disease
CVC: cardiovascular continuum
